# Characterizing the Health of Older Rural Australians Attending Rural Events: Implications for Future Health Promotion Opportunities

**DOI:** 10.3390/ijerph19053011

**Published:** 2022-03-04

**Authors:** Tracy L. Schumacher, Laura Alston, Luke Wakely, Rachel Latter, Kelly Squires, Susan Heaney, Leanne J. Brown

**Affiliations:** 1 Department of Rural Health, University of Newcastle, Tamworth, NSW 2340, Australia; tracy.schumacher@newcastle.edu.au (T.L.S.); luke.wakely@newcastle.edu.au (L.W.); kelly.squires@newcastle.edu.au (K.S.); susan.heaney@newcastle.edu.au (S.H.); 2 Hunter Medical Research Institute, New Lambton Heights, NSW 2305, Australia; 3 Research Department, Colac Area Health, Colac, VIC 3707, Australia; laura.alston@deakin.edu.au; 4 The Global Obesity Centre, Institute for Health Transformation, School of Medicine, Faculty of Health, Deakin University, Geelong, VIC 3220, Australia; 5 Deakin Rural Health, Deakin University, Warrnambool, VIC 3280, Australia; 6 St. George Hospital, South Eastern Sydney Local Health District, Kogarah, NSW 2217, Australia; rachel.latter@health.nsw.gov.au

**Keywords:** rural health, nutrition, health promotion, health status, older adults

## Abstract

This paper describes the health of older Australians (>65 years) attending rural events to inform health promotion interventions for rural populations. This cross-sectional study collected survey data and objective health measures between 2017 and 2020 at two events held in rural New South Wales, Australia. Participants included in the analysis were adults > 65 years of age. Data included demographic and health information, anthropometric measures (height, weight, waist circumference), and dietary and physical activity data. A total of 256 people > 65 years participated. Our sample, which was mostly male (59.0%), contained people aged between 66 and 75 years (72.3%). Participants lived in either a large rural (34.0%) or small rural town (22.3%), with low levels of education (60.9% did not complete high school). Dietary quality was rated as below average. All but 17.2% of the participants reported having a health condition. The risk of a health condition was associated with increasing age, lower education, and higher waist circumference, but not remoteness. Rural events may provide an opportunity to access, engage with, and understand the health of older rural Australians, especially males. They may offer ideal contexts for health and nutrition promotion opportunities in rural areas where access to health professionals is limited.

## 1. Introduction

Older individuals make up a considerable proportion of Australia’s population, with more than one in seven people being aged 65 years and over [1]. It is estimated that by 2022, four million Australians will be aged 65 and over, which is expected to be followed by a rapid increase in the proportion of this age group over the next decade [2]. For this group, the World Health Organization (WHO) (Geneva, Switzerland) definition of healthy ageing as “the process of developing and maintaining the functional ability that enables wellbeing in older age” applies [3]. Chronic diseases become increasingly prevalent and can significantly impact economic, social, and physical aspects of quality of life [4]. A focus on health promotion for the generation and maintenance of optimal health and nutrition in an ageing population is key to an improved quality of life and a reduced impact on health services in the future.

One in two people aged 65 years and over are diagnosed with one or more chronic diseases, such as cardiovascular disease, cancer, diabetes, chronic kidney disease, and mental health conditions [4,5]. Over one-third of disease burden could be prevented by reducing or eliminating exposure to modifiable risk factors, including poor nutrition, physical inactivity, smoking, and harmful use of alcohol [6,7,8]. Where people live may influence exposure to modifiable risk factors and, therefore, can further increase the risk of developing chronic disease [9]. In addition, health and risk factor data for those living in rural areas in Australia are limited, resulting in a lack of evidence-informed action and policy [10,11].

Older Australians are less likely to live in major cities compared to the general population, with one-third of older Australians living in regional or remote areas in 2016 [1]. Rural and remote areas of Australia can be classified by the Modified Monash Model which takes into account population size and remoteness of the geographical area as well as access to services [12]. These areas tend to be heterogenous in nature; however, on average, they have poorer health outcomes compared to metropolitan communities [13,14]. The reasons for this are complex and multifactorial [13,14,15,16]; however, the health inequities have been widely acknowledged to be unjust and preventable [10,17]. Issues such as geographical isolation, limited access to health services, lower education and health literacy levels, differences in modifiable risk factors, lower socioeconomic status, and a lack of evidence to inform health policy, compared to metropolitan communities, contribute to these health outcomes for older people in these areas [9,10,13,14,15,16,18]. Rurality has been shown to be a risk factor for chronic diseases, over and beyond the influence of factors such as socio-economic status [19]. Health inequalities in the burden of chronic diseases among rural Australians are well documented in the Australian literature; however, health promotion initiatives to date have often failed to address the unique needs of rural communities [17,20].

Despite the known health inequalities experienced by rural Australians, a recent review found that there is only limited evidence of nutrition interventions in rural areas, with an urgent need for further research in rural communities [21]. Rural people are under-represented in existing health data sets [22] and due to their geographical location are more challenging to access and engage in health promotion opportunities [23,24]. Previous health-related activities have been conducted at a rural agricultural events in Australia, with one specifically focused on the health of men who self-presented to a health promotion activity [25]. Most participants in this study were rural-based, with a median age of 56 years and had ‘at risk’ health characteristics [25].

Large rural events offer an opportunity to obtain health data and to promote health improvements in this population [25]. Many of these events intend to bring geographically isolated communities together to showcase agricultural practices, produce products, and/or to promote tourism which provides social and economic benefits [26,27,28]. There are many rural-based events across Australia each year and they may appeal to different groups with variations in the age, gender, and rurality of those attending. These events provide an opportunity to engage with these populations, with access to healthy and ‘at risk’ rural people to provide health and nutrition promotion [25].

Health promotion initiatives may alleviate the increased social and economic burden in Australia and globally due to the growing ageing population and concomitant chronic disease rates [2]. Targeted health promotion for older rural people may support successful ageing with an increased interest in health promotion programs for this target group [24]. Internationally, researchers have found that health promotion is challenging in rural communities, with practitioner, client, and site factors restricting what can be done [23,24]. Opportunities exist to provide health and nutrition promotion at events attended by large numbers of rural people.

### The CHAaRGE:20 Study

The Changing Health Actions at Rural and Regional Events in 20 min (CHAaRGE:20) is a health promotion initiative that aims to address health and nutrition issues for populations attending rural-based events. The CHAaRGE:20 stall delivers 20 min health checks at rural-based events and provides participants with individualized feedback (brief nutrition and health promotion) on their health and nutrition measures. During these health checks, anthropometric measurements are taken, and health and demographic data are collected to increase the understanding of the health issues prevalent in the population. Over the various events, a range of health measures have been trialed as practical and useful approaches for opportunistic health promotion. This larger data set has informed the development of brief nutrition and health promotion materials, along with changes to the health check assessment components.

In order to develop appropriate health and nutrition interventions for rural people, a greater understanding of their health status and health promotional tools is needed. Older rural Australians can be supported to improve their health as they age, with consideration of the individual and collective factors that impact on their wellbeing. A greater understanding of ways to connect and engage with them are key to supporting improvements through health- and nutrition-focused promotional activities [29]. This is a novel study as it utilizes existing events where rural people gather in large numbers to collect data about their nutrition and health status in order to inform future health promotion that is targeted to suit their needs and stage of life. Understanding the health and nutrition issues of older rural Australians will support initiatives to improve their health with age.

The aim of this paper is to characterize the health and nutrition of older Australians (>65 years) that attended a free health promotion stall in two rural sites to inform future health promotion opportunities.

## 2. Materials and Methods

This was a cross-sectional study with data collected between 2017 and 2020 at two regional event sites in New South Wales (NSW), Australia. 

### 2.1. Participants and Setting

Adults aged ≥ 18 years were eligible for the health check activity. Participants needed to be able to read and follow instructions in English and physically able to stand for height and weight measures. However, only people providing their age as over 65 years were included in this analysis, with the full data set for all age groups reported elsewhere in an analysis of diet quality [30]. Attendees under the influence of drugs or alcohol were considered ineligible. Recruitment was undertaken at two event sites: (i) AgQuip, a three-day agricultural field day based in Gunnedah, NSW in August (2017–2019); and (ii) the Tamworth Country Music Festival (TCMF), held in January (2018–2020). Data for AgQuip were typically collected for the entire three-day period between 9:00 a.m. and 4:00 p.m. every day at the University of Newcastle Department of Rural Health (UONDRH) site at the AgQuip field day. The TCMF site was located within an air-conditioned shopping complex, although complexes changed between 2018 and 2019/2020. Both complexes had access to locals and visitors attending the events, and data were typically collected between 9:00 a.m. and 4:00 p.m. Although the TCMF officially runs between mid- and late-January annually, the study only recruited at the site for three or four days (Monday–Thursday) during the ten-day festival.

Participants were recruited opportunistically at the events. Study information flyers were distributed by student volunteers, UONDRH social media (Facebook and Twitter), local media, word of mouth, and direct participant enquiry at the UONDRH health exhibition. The stall was staffed by UONDRH academic health professional staff and students. Qualified health professionals and students with training in the study recruitment and data collection processes were able to perform aspects of the research process.

Ethical approval was obtained from the University of Newcastle Human Research Ethics Committee (H-2017-0197) and was conducted in accordance with the National Statement on Ethical Conduct in Human Research. Informed consent was obtained prior to the collection of data and measurements. 

### 2.2. The Health Check Activity

Data collection took approximately 20 min per participant. The survey and health measurements were undertaken as part of a health check where participant privacy was provided using large screens within an open space area. Each participant was provided with a participant information statement and was able to complete their consent to participate on an iPad. Participants were allocated a unique study identification number. At the end of the health check, participants were provided with their health assessment results and a comparative recommended or average score.

### 2.3. Demographic and Health Survey

A demographic and health survey was completed by participants on iPads provided and participants were assisted by student dietitians or researchers if needed. Key demographic and health information collected included residential location, measures of socioeconomic status, education and employment, smoking status, and dental health and medical history. Australian Physical Activity Guidelines were used to compare cohort physical activity data for AgQuip 2019 onwards [31]. All questions consisted of multiple-choice options. The survey was managed using the online Survey Monkey program in 2017 and Research Electronic Data Capture (REDCap) software in 2018 [32]. Some categories with small numbers of respondents were collapsed.

### 2.4. Geographic Location

Rurality was categorized into the Modified Monash Model [12], which provides a classification of Australian cities and towns from Modified Monash (MM) 1 to 7: MM1—metropolitan, MM2—regional centers, MM3—large rural towns, MM4—medium rural towns, MM5—small rural towns, MM6—remote communities, and MM7—very remote communities. By using postcode, the description of where someone lived (in town/out of town/don’t wish to answer), the size of the nearest town (<5000/500–15,000/15,000–50,000/50,000 + people), and the size of their land if the “out of town” descriptor was chosen (large house block/hobby farm/small farm/medium farm/large farm/don’t wish to answer). A postcode with a single MM categorization was given that categorization, regardless of any further information provided. Some postcodes had more than one MM categorization e.g., the postcode 2340 may be categorized as MM3 (large rural town) or MM4 (medium rural town). When further information was provided, i.e., with answers of “in town”, “large house block”, “hobby farm”, “small farm”, and “medium farm”, these were attributed to the MM value for that postcode and town size. Those choosing “out of town” and “large farm” were attributed an MM code consistent with being 20 km from a town within that postcode. Where limited information was available, or other factors made the MM attribution unclear, the lowest MM value for that postcode was given.

### 2.5. Dietary Intake

Dietary intake was assessed using the online Healthy Eating Quiz [33,34] at the health checks undertaken in 2017. In 2018, this was changed to the optional completion of the Australian Eating Survey (AES) [35], as quiz completion was slower than expected. Within the survey, participants had the option to provide their email address and to complete the AES online at a later time. An automated email was sent using REDCap with a link to the AES being delivered to consenting participants upon completion of the demographic and health survey. A reminder was sent approximately 1–2 weeks post-event for those who had not completed the survey or if their survey was incomplete. The AES is a detailed dietary questionnaire designed to collect information regarding an individual’s usual intake and dietary patterns. Both the Healthy Eating Quiz and AES generate the same diet quality score for the Australian Recommended Food Score (ARFS) [36]. The ARFS ranges from 0 to 73, and questions align with foods in the core food groups of The Australian Guide to Healthy Eating [37]. Scores are categorized as “needs work” (<33), “getting there” (33–38), “excellent” (39–46), or “outstanding’ (47+)”.

### 2.6. Anthropometry

Anthropometry measures were recorded by trained research assistants according to International Society for the Advancement of Kinanthropometry (ISAK) protocols. A portable BSM370 stadiometer, corrected to 0.1 cm and 0.01 kg (Biospace Co., Ltd., Seoul, Korea), was used to measure participant height and weight. Waist circumference was measured on the skin using a metallic, non-extensible Lufkin WP606PM tape measure at the narrowest point between the lower costal border and the iliac crest. Waist circumference was categorized as increased risk at 80 cm for women and 94 cm for men, and greatly increased risk at 88 cm and 102 cm for women and men, respectively [38]. Prior to all anthropometric measures, participants were asked to remove their shoes and any heavy items on their person such as belt, watch and/or phones. Measures were recorded a minimum of two times for each participant and the average of these was recorded. In cases where variation in measures was > 2%, an additional measure was taken. An average was calculated from the two closest measures. Body mass index (BMI) was calculated as kg/m^2^ and categorized into ‘underweight, healthy weight, overweight and obese’ [39]. From 2018, participants were also asked to estimate their own weight. This was divided by their actual weight and multiplied by 100 to calculate the percentage of actual weight that was estimated. 

### 2.7. Statistical Analysis

Data were managed using Survey Monkey in 2017 and REDCap from 2018 onwards and analyzed using Stata/IC Version 15.1 (StataCorp. 2017. Stata Statistical Software: StataCorp LLC, College Station, TX, USA). Descriptive statistics were used to describe participant demographics and health characteristics, with normally distributed variables presented as mean ± standard deviation (SD). Percentages of respondents were given where possible. A Kruskal–Wallis equality of populations was used to test equality in health characteristics between those who responded to the diet questionnaire, and those who did not. A linear regression was used to test whether rurality contributed to a person’s total number of self-reported health conditions. Health conditions (high blood pressure or high cholesterol, renal disease, diabetes, cancer, mental health, musculoskeletal conditions, or respiratory conditions) were self-reported, based on if they had been told they had this by a health professional. An adjusted model contained contributing factors that were decided a priori, based on literature and whether they had been consistently collected over the recruiting period. Therefore, rurality classified by MMM, age, gender, education, and waist circumference were included in the model. Using *pmsampsize* (Riley 2020) and based on an r-squared value of 0.1, five parameters, an intercept of 2, and a sample size of 239 people were needed.

## 3. Results

A total of 638 participants completed all the required components of the study. Of these, *n* = 256 were aged over 65 years of age (therefore *n* = 382 was excluded). No people were turned away for not meeting the inclusion criteria. Participant demographic information for these people is summarized in Table 1.

Risk factors for chronic disease, such as weight status, smoking, physical activity, and dietary estimates, can be seen in Table 2. It shows few differences between people attending the two different rural events. According to World Health Organization BMI categorization, the majority of people were overweight (*n* = 117, 45.7%), with 33.2% classed as obese (*n* = 85), and the remainder as normal weight (*n* = 54, 21.1%). BMI was 29.4 kg/m^2^ (4.0) for men and 26.6 kg/m^2^ (4.4) for women. Men weighed a mean of 88.8 kg (12.0), while women weighed 68.7 kg (12.4). Of the 68 people (26.6%) categorized as “increased risk” according to their waist circumference, the distribution was similar for men and women (27.2% and 25.7%, respectively). However, of the 140 (54.7%) with a waist circumference of “greatly increased risk”, males represented more of the sample (59.6% males and 47.6% females). Waist circumference was 105.1 cm (12.6) for men and 87.8 cm (11.7) for women. Across the total sample, 14.8% stated they had recently lost weight.

It must be noted that most people were accurate in estimating their weight. With 100% representing complete accuracy, the median accuracy was 98.9%, with an interquartile range of 97.5% to 100.6%. Approximately 6.2% of the sample population estimated a weight more than 5% lower than their measured weight, and only 2.7% provided an estimation more than 5% above their measured weight. There was no statistical difference in accuracy between gender (*p* = 0.32) or event (*p* = 0.94).

Of the 161 participants who were asked about their physical activity, the preferred method of physical activity was walking, with 74.7% of the TCMF participants and 84.1% of AgQuip participants performing this activity for one hour or more each week. Despite 85.7% of respondents answering that they are active on most days, only 52.8% also said they felt they got enough physical activity.

On average, the proportion of energy from energy dense, non-nutritious foods was 30%. The Healthy Eating Quiz was completed by 23 participants, prior to changing the dietary measure to the optional AES, which was completed by 54 participants. The mean ARFS score was 36.2 (SD 8.5) (*n* = 77), which rates as ‘getting there’, with no difference between events. Of those who completed the optional AES, the average energy contributed by core foods was 69.9% of total intake. 

Oral health and reported health conditions can be found in Table 3. Although oral health data were not available for the 2017 AgQuip period, it shows that the majority of the population retains all or some of their own teeth. 

On average, participants 1.8 (SD 1.2) health conditions and took medication for 0.93 (SD 0.83) for those conditions. Notably, approximately half of those who chose to undertake a health check (*n* = 22, 54.4%) were on a pension. The number of health conditions and BMI did not differ between those who had completed the dietary questionnaire and those who did not (*p* = 0.085 and *p* = 0.352 respectively).

Rurality did not contribute to the number of health conditions a person reported they had, as advised by a health professional (*p* = 0.194), although age, education, and waist circumference all had significant associations (see Table 4). In this sample, the number of health conditions increased by 0.2 for each year of age (95% confidence interval (CI): 0.050–0.345). Each higher level of education decreased health conditions by approximately 0.18 (95% CI: −0.318–−0.040), and each centimeter of waist circumference increased health conditions by 0.016 (95%CI: 0.006–0.025).

## 4. Discussion

This paper characterizes the health of a sample of largely rural dwelling Australians over 65 years of age, a population that has been historically under-researched resulting in minimal evidence to inform health promotion priorities for this age group [40]. Of most interest was that our population were predominantly male (59%), which addresses important gaps in the evidence as, broadly speaking, males tend to be difficult to recruit in health research, especially in rural areas [41,42]. Overall, most of the population (72.3%) were aged between 66 and 75 years of age and lived in either a large rural (MM3 34.0%) or small rural (MM5 22.3%) town, with lower levels of education (most of our sample reported that they had not completed high school (60.9% of participants)). All but 17.2% of the participants reported that they had a health condition, and the risk of a health condition was associated with increasing age, lower education status, and higher waist circumference, consistent with the wider literature [4,5]. Having a health condition was not found to be associated with remoteness in this sample, in comparison to larger Australian studies that have been inclusive across a wider age range [17,19]. This may be due to the smaller and more male-dominated sample analyzed in this context, which is novel compared to previous research.

There is a need for ongoing health promotion in rural areas with a focus on education for improved nutrition, healthy weight, good oral health, and the management of chronic health conditions. Improving nutrition is key to the optimization of health for the elderly who live in rural areas, as it can support weight loss and assist in management of chronic conditions. Key messages for dietary change are to achieve more variety from core food groups and to reduce energy dense nutrient poor foods in order to improve overall diet quality.

Our data were largely reflective of estimates from national health surveys in Australia, with most of the participants being in the overweight and obese category (78.9%) compared with the national estimate of 75.6% for this age group [1,43]. In contrast, only a small number (1–3%) of this sample reported being a current smoker compared to 6–7% of over-65-year-olds in national data [1]. This may be due to selection bias, with the opportunistic nature of the health checks and the likelihood that people attending wanted to know more about their health and are more likely to engage in more healthful behaviors as a result [44]. Our findings support the existing literature for this age group, i.e., that walking followed by housework and gardening are the most preferred forms of physical activity. This is consistent with another large Australian study that also investigated barriers and facilitators to physical activity in older people [45]. Similarly, our population identified pain as a main barrier to exercise (27.6% in the current study compared to 26% previous estimates) with both time and tiredness also being reported as barriers, albeit time being a greater barrier in this current study (30%) and only 12% in the previously published study [45]. Most participants (78.9%) reported that they engaged in the recommended 150 to 300 min of exercise per week. Conversely, national data describe that 71.9% of those aged 65 years and over do not meet the recommended level of physical activity [43]. This may again be reflective of selection bias in our study, where more health interested participants are more likely to attend the health checks and participate in research [43].

Despite the high proportion of overweight and obesity in this sample, 14.8% reported that they had recently lost weight. Our study did not explore the reasons behind this, and whether the weight loss was intentional. This is an important consideration for future research, as there is evidence to suggest that rural-dwelling Australians are at an increased risk of malnutrition, especially in the over-65-year-old age group [46]. Furthermore, participants reported that a high proportion of their daily energy intake was from non-nutritious energy dense foods, which may both contribute to overweight and obesity, alongside the risk of malnutrition [47]. This is important as both overweight, obesity, and malnutrition contribute to poorer health outcomes, making this population especially vulnerable [47]. Globally, clinical guidelines have attempted to take this into account, through debate over what is considered the ‘healthy weight range’ for those aged over 65 years. Guidelines for clinical settings suggest that the ideal BMI range for older people is 27–30 kg m^2^, arguing that increased weight is protective in the event of acute illness [48]. Despite this, national data for this age group are reported alongside the WHO guidelines of a BMI of >25 m^2^ as being considered overweight or obese. With most of our sample reporting that they have existing health conditions, consideration around the healthy BMI range is needed, and should be explored in future health promotion initiatives. Perhaps reflective of this lack of consistency, only 16% of participants had been told by their doctor that they were overweight or obese, despite a high proportion in our sample. This may also be indicative of the lack of access to general practitioners and health professionals in rural settings, reducing the opportunity for such discussions to take place [9,49,50].

Importantly, there is a paucity of literature focused on interventions to improve dietary behaviors in rural areas [21]. Our sample, with an average ARFS of 36.2 (SD 8.5), had comparable diet quality scores with a larger sample of Australian adults aged 65–74+ years, where ARFS ranged between 35.8 (15.3) and 37.2 (SD 8.5) [33]. This equates to a diet quality categorized as ‘getting there’. In our previously reported comparison of diet quality with CHAaRGE:20 participants of other age groups, those aged over 71 years had higher scores for diet quality compared to 18–30-year-old participants [30]. Several risk factors that relate to lower diet quality were reported here. For example, previous research has shown that living alone is one such risk factor [30,33]. Approximately one third of this sample lived alone, and is consistent with other populations [1]. Our study similarly showed that the oral health reported here is also consistent with national data, such as having a dry mouth and sore spots, and this may affect nutritional intake [1,51]. The relationship between diet quality and chronic diseases is well known [52]. Risk factors related to nutrition require further consideration when tailoring health promotion initiatives specifically for this age group.

This study shows that such events provide an ideal setting for health promotion initiatives which aim to improve health. Brief nutritional interventions have been shown to have a positive impact on short-term dietary behaviors, particularly if education and instructional component are tailored to individuals [53]. Targeted nutrition interventions, focused on fruit and vegetable intake, fat intake, and/or diet quality, included a consultation with personalized feedback, as opposed to generic nutrition information, which resulted in a positive improvement in dietary change (low fat dairy intakes at 1-month follow-up) at 6 and 12 months [53].

Older Australians are more likely to live in rural and remote areas and are vulnerable to health inequalities observed between differing levels of remoteness than people aged under 65 [9,20]. Recruitment of older adults at these rural events was higher than the relative proportion of those over 65 years living outside of an Australian capital city [54]. Our study shows that rural events are a useful setting for recruiting rural and remote Australians over the age of 65, especially males. In particular, events focused on agriculture may provide a valuable opportunity to engage with males, as our study achieved a higher proportion of male participation at this type of event. Our study provided an ideal opportunity for gaining new understandings and implementing health promotion initiatives. In this study, we were able to easily recruit a large sample, with a high response rate to most questions. Most of the participants also indicated that they visited the health check because they wanted to know more about their health, which again could be reflective of a lack of access to health services in rural settings [17]. This study highlights the opportunities rural events offer in collecting data that advances understandings of the health of people in non-metropolitan areas. Rural events also offer an opportunity for brief health promotion and education initiatives, as most participants were not highly educated but were motivated to know more about their health. 

Although our study presents comprehensive data on a historically understudied population, it is evident that further research is needed with this age group. Investment in health promotion initiatives that consider a focus on addressing weight in this group, balancing the risk of malnutrition, and providing advice around managing pain and exercise is needed. This study also provides evidence that there is a need for health promotion initiatives that target both the prevention and management of oral health issues. Future research also needs to consider how rurally based health professionals can better approach conversations around weight status in this age-group, as well as how this can be appropriately addressed to reduce the risk of new or worsening non-communicable disease in this age group.

A strength of this study is it provides a data set on an under-researched population that used survey tools based on national data collection questions and objectively measured weight and height by trained health researchers. The data were also collected at two separately located events over four years, adding to the strength and generalizability of the dataset. A potential limitation to this data is that the people attending the health check may have been more health-conscious, therefore presenting different conditions and behaviors compared to the general population. This particularly extends to those with dietary data obtained from completed AES. This study also relied on self-report for most of the data collection, which is also subject to reporting bias. However, our data largely reflected findings from recent national surveys, indicating representativeness. Questions and subsequent data collection evolved over time, and this has meant limited responses in some areas. A further limitation is that changing to voluntary completion of the AES limited the number of dietary data observations, but increased the level of detail. Additionally, in the absence of any further refining information, participants were categorized with their lowest relevant MMM. This meant that some participants who may have resided in a higher MMM, or more rural area, were actually classified as living in a more populous region. 

## 5. Conclusions

Our data provide evidence that rural events provide an ideal opportunity for collecting data on rural Australians aged over 65 years, particularly for males who are under-reported in the literature. Our findings also show that there is a need for further research into opportunistic health promotion initiatives that can be applied at these events and that offer education on nutrition, healthy weight, oral health, and managing health conditions by considering the specific needs of this age group.

## Figures and Tables

**Table 1:** CHAaRGE:20 study participants (aged over 65 years) demographic information by rural event.

		AgQuip		TCMF		Total	
		*n* = 105	(100%)	*n* = 151	(100%)	*n* = 256	(100%)
**Age**							
	66–70 years	38	(36.2%)	61	(40.4%)	99	(38.7%)
	71–75 years	40	(38.1%)	46	(30.5%)	86	(33.6%)
	76–80 years	20	(19.1%)	27	(17.9%)	47	(18.4%)
	81 + years	7	(6.7%)	17	8.6%)	24	(9.4%)
**Gender**							
	Male	74	(70.5%)	77	(51.0%)	151	(59.0%)
	Female	31	(29.5%)	74	(49.0%)	105	(41.0%)
**MM rural categorization** **(live in or near)**							
	1—metropolitan area	12	(11.4%)	45	(29.8%)	57	(22.3%)
	2—regional centre	2	(1.9%)	10	(6.6%)	12	(4.7%)
	3—large rural town	24	22.9%)	63	(41.7%)	86	(34.0%)
	4—medium rural town	24	(22.9%)	7	(4.6%)	31	(12.1%)
	5—small rural town	37	(35.2%)	20	(13.3%)	57	(22.3%)
	6–7—remote–very remote community	3	(2.9%)	1	(0.7%)	4	(1.6%)
	Missing	3	(2.9%)	5	(3.3%)	8	(3.1%)
**Education**							
	Less than year 12	60	(57.1%)	96	(63.6%)	156	(60.9%)
	Year 12	8	(7.6%)	18	(11.9%)	26	(10.2%)
	Trade or vocation	18	(17.1%)	25	(16.6%)	43	(16.8%)
	University or above	17	(16.2%)	10	(6.6%)	27	(10.6%)
	None above/missing/don’t wish to answer	2	(1.9%)	2	(1.3%)	4	(1.6%)
**Household income**							
	Pension	33	(31.4%)	43	(28.5%)	76	(29.7%)
	AUD 0–499/week	18	(17.1%)	30	19.9%)	48	(18.8%)
	AUD 500–999/week	14	(13.3%)	19	(12.6%)	33	(12.9%)
	AUD 1000–1999/week	11	(10.5%)	25	(16.6%)	36	(14.1%)
	AUD 2000+/week	6	(5.7%)	3	(2.0%)	9	(3.5%)
	Don’t know/wish to answer	23	(21.9%)	31	(20.5%)	54	(21.1%)
**People dependent on this income**							
	1	39	(37.1%)	67	(44.4%)	106	(41.4%)
	2–3	59	(56.2%)	77	51.0%)	136	(53.1%)
	4–6	3	(2.9%)	2	(1.3%)	5	(2.0%)
	Don’t wish to answer/missing data	4	(3.8%)	5	(3.3%)	9	(3.5%)
Living arrangements *							
	Live alone	29	(27.6%)	50	(33.1%)	79	(30.9%)
	With partner/spouse	71	(67.6%)	94	(62.3%)	165	(64.5%)
	Own children or other’s children	5	(4.8%)	10	(6.6%)	15	(5.9%)
	Other adults	6	(5.7%)	7	(4.6%)	13	(5.1%)

Abbreviations: AUD-Australian Dollars; MM—Modified Monash; TCMF—Tamworth Country Music Festival. * Note that people were able to tick more than one box with this question.

**Table 2:** Description of risk factors of CHAaRGE:20 study participants (aged over 65) attending the rural events.

		AgQuip		TCMF		Total	
**Smoking status**		***n* = 105**	**(100%)**	***n* = 151**	**(100%)**	***n* = 256**	**(100%)**
	Currently smoke	1	(1.0%)	3	(2.0%)	4	(1.6%)
	Do not currently smoke	104	99.1%)	147	(97.4%)	251	(98.1%)
	Missing data	0	(0%)	1	(0.7%)	1	(0.4%)
**Weight status** (*n* = 256)		*n* = 105	(100%)	*n* = 151	(100%)	*n* = 256	(100%)
**Weight over the last 6 months**							
	Been losing or have lost weight	15	(14.3%)	23	(15.2%)	38	(14.8%)
	Maintained weight	55	(52.4%)	91	(60.3%)	146	(57.0%)
	Gained weight	12	(11.4%)	32	(21.2%)	44	(17.2%)
	Not sure or missing data	23	(21.9%)	5	(1.3%)	28	(10.9%)
		**mean**	**(sd)**	**mean**	**(sd)**	**mean**	**(sd)**
	Est. weight (kg, *n* = 224, n/a AgQuip 2017)	84.5	(19.0)	77.6	(17.1)	80.0	(18.1)
	Measured weight (kg, *n* = 255)	83.8	(16.0)	78.2	(17.6)	80.5	(17.1)
	Measured BMI (*n* = 255)	28.9	(3.9)	27.8	(4.7)	28.2	(4.4)
	Measured waist circumference (cm)	100.5	(14.2)	97.0	(15.7)	98.4	(15.2)
Physical Activity * (those answering ‘*yes*’)		***n* = 82**	**(100%)**	***n* = 79**	**(100%)**	***n* = 161**	**(100%)**
	Do you feel you participate in enough physical activity?	45	(54.9%)	40	(50.6%)	85	(52.8%)
	Active on most days of every week	70	(85.4%)	68	(86.1%)	138	(85.7%)
	Moderate (150–300 min) or vigorous activity (75–150 min)/week	60	(73.2%)	67	(84.8%)	127	(78.9%)
	Muscle strengthening at least 2 days/week	32	(39.0%)	27	(34.2%)	59	(36.7%)
**Activities for 1 or more hours in the last week**							
	Physical activity, e.g., swimming, jogging, tennis, gym	14	(17.1%)	20	(25.3%)	34	(21.1%)
	Cycling	7	(8.5%)	7	(8.9%))	14	(8.7%)
	Walking	69	(84.1%)	59	(74.7%)	128	(79.5%)
	Housework	41	(50.0%)	43	(54.4%)	84	(52.2%)
	Gardening/Do It Yourself activities	36	(43.9%)	39	(49.4%)	75	(46.6%)
Barriers to physical activity *		*n* = 37	(100%)	*n* = 39	(100%)	76	(100%)
	Pain, injury or illness	10	(27.0%)	11	(28.2%)	21	(27.6%)
	Too tired	3	(8.1%)	6	(15.4%)	9	(11.8%)
	No easy access	4	(10.8%)	4	(10.3%)	8	(10.6%)
	No time	12	(32.4%)	11	(28.2%)	23	(30.3%)
	Too expensive	1	(2.7%)	3	(7.7%)	4	(5.3%)
	Weather makes physical activity difficult	0	(0.0%)	9	(23.1%)	9	(11.8%)
	Lack of trained people to show how to participate	1	(2.7%)	1	(2.6%)	2	(2.6%)
	Unsure of how to participate	3	(8.1%)	6	(15.4%)	9	(11.8%)
**Diet**		**mean**	**(sd)**	**mean**	**(sd)**	**mean**	**(sd)**
	ARFS (*n* = 77, AgQuip = 41/TCMF = 36) ^	35.5	(8.5)	36.9	(8.6)	36.2	(8.5)
	% energy from recommended food sources (*n* = 54, AgQuip = 18/TCMF = 36)	71.2	(11.0)	69.3	(11.2)	69.9	(11.1)
	% energy from energy dense, nutrient poor foods (*n* = 54, AgQuip = 18/TCMF = 36)	28.8	(11.0)	30.8	(11.3)	30.1	(11.1)

The number of responses varied according to when questions were added to surveys used. Therefore, *n* values are presented for each section. * Physical activity questions added to intervention in prior to AgQuip 2019. Only people who answered NO to whether they participated in enough physical activity were asked about barriers. ^ No significant difference in Australian Recommended Food Score (ARFS) between those completing the Healthy Eating Quiz and Australian Eating Survey; Abbreviations: ARFS—Australian Recommended Food Score; BMI—body mass index; Est—estimated; TCMF—Tamworth Country Music Festival.

**Table 3:** Description of self-reported health conditions and medications, as identified by self or told by a health practitioner.

Self-Reported Health Information			AgQuip		TCMF		Total	
			*n* = 105	(100%)	*n* = 151	(100%)	*n* = 256	(100%)
**Oral Health**								
	Care for own teeth		79	(75.2%)	146	(96.7%)	225	(87.9%)
	Have a dry mouth							
		Always	5	(4.8%)	12	(8.0%)	17	(6.6%)
		Sometimes	33	(31.4%)	68	(45.0%)	101	(39.5%)
	Have sore spots in your mouth							
		Always	1	(1.0%)	2	(1.3%)	3	(1.2%)
		Sometimes	4	(3.8%)	12	(8.0%)	16	(6.3%)
		Infrequently	3	(2.9%)	7	(4.6%)	10	(3.9%)
	Have own teeth							
		Own teeth	46	(43.8%)	67	(44.4%)	113	(44%)
		Combination own teeth/dentures	30	(28.6%)	62	(41.1%)	92	(35.9%)
		Full set of dentures	5	(4.7%)	19	(12.6%)	24	(9.4%)
		Missing data	24	(22.9%)	3	(2.0%)	27	(10.6%)
**Health conditions ^**								
	High blood pressure or high cholesterol		52	(49.5%)	83	(55.0%)	135	(52.7%)
	Chronic kidney or renal disease		4	(3.8%)	2	(1.3%)	6	(2.3%)
	Diabetes (Type 1, 2 gestational or other)		12	(11.4%)	17	(11.3%)	29	(11.3%)
	Overweight or obesity		23	(21.9%)	18	(11.9%)	41	(16.0%)
	Cancer (any type)		26	(24.8%)	22	(14.6%)	48	(18.8%)
	Anxiety, depression, schizophrenia or any other mental health condition		14	(13.3%)	16	(10.6%)	30	(11.7%)
	Back problems, osteoporosis, osteoarthritis and rheumatoid arthritis or any other musculoskeletal condition		49	(46.7%)	68	(45.0%)	117	(45.7%)
	Asthma, COPD or any other lung condition		19	(18.1%)	24	(15.9%)	43	(16.8%)
	None of these conditions		17	(16.2%)	27	(17.9%)	44	(17.2%)
**Take regular medications for these health conditions**								
	High blood pressure or high cholesterol		49	(53.3%)	77	(49.0%)	126	(49.2%)
	Chronic kidney or renal disease		3	(2.9%)	0	(0.0%)	3	(1.2%)
	Diabetes		9	(8.6%)	14	(9.3%)	23	(9.0%)
	Overweight or obesity		0	(0%)	0	(0%)	0	(0%)
	Cancer		1	(1.0%)	0	(0%)	1	(0.4%)
	Anxiety, depression, schizophrenia or any other mental health condition		4	(3.8%)	8	(5.3%)	12	(4.7%)
	Back problems, osteoporosis, osteoarthritis and rheumatoid arthritis or any other musculoskeletal condition		15	(14.3%)	34	(22.5%)	49	(19.1%)
	Asthma, COPD or any other lung condition		7	(6.7%)	17	(11.3%)	24	(9.4%)
**Reason for the health check**								
	Because it was free		20	(19.1%)	22	(14.6%)	42	(16.4%)
	I wanted to find out more about my health		44	(41.9%)	48	(31.8%)	92	(35.9%)
	It’s been a while since I had a health check		11	(10.5%)	5	(3.3%)	16	(6.3%)
	My partner/spouse/friend etc. said I should		10	(9.5%)	7	(4.6%)	17	(6.6%)
	I wanted to help with research about rural health		20	(19.1%)	17	(11.3%)	37	(14.5%)

Only those who answered YES are described in the table. ^ Question asked was “Have you ever been told by a doctor or health professional that you have any of these conditions?” Abbreviations: chronic obstructive pulmonary disease—COPD; TCMF—Tamworth Country Music Festival.

**Table 4:** Unadjusted and adjusted regression results for whether rurality is a significant contributor to the number of health conditions a person reported they had been told by a health professional. Model fit for the adjusted regression was *p* < 0.001 and adjusted R-squared was 0.1002.

	Coefficient	Std. Err.	*p* Value	95% Confidence Interval
MMM (unadjusted)	0.095	0.053	0.072	−0.008–0.199
MMM (adjusted)	0.072	0.075	0.178	−0.033–0.350
Age	0.202	0.075	0.008	0.053–0.345
Gender	0.173	0.196	0.378	−0.213–0.558
Education	−0.158	0.070	0.025	−0.296–−0.203
Waist circumference	0.016	0.006	0.032	0.006–0.031

Abbreviations: MMM—Modified Monash Model.

## Data Availability

Data may be available upon request to the corresponding author Leanne Brown (Leanne.Brown@newcastle.edu.au). Each request will be considered on a case-by-case basis.
